# Carotid Webs: An Unusual Presentation of Fibromuscular Dysplasia

**DOI:** 10.7759/cureus.9549

**Published:** 2020-08-04

**Authors:** Shivani Priyadarshni, Anamika Neralla, Joseph Reimon, Shaun Smithson

**Affiliations:** 1 Internal Medicine, Aventura Hospital and Medical Center, Aventura, USA; 2 Cardiology, Aventura Hospital and Medical Center, Aventura, USA

**Keywords:** extracranial fibromuscular dysplasia, renal fibromuscular dysplasia, carotid webs, arterial dissections, arterial thrombus occlusion, endovascular and surgical therapy, vertebral arteries, right internal carotid artery, pseudoaneurysm, percutaneous transluminal balloon angioplasty

## Abstract

Carotid webs are abnormal luminal projections at the carotid bulb associated with blood flow stasis, artery dissection, and subsequent complications. Carotid webs are considered to be a rare variant of fibromuscular dysplasia (FMD). Young individuals with symptomatic carotid webs are found to be associated with ischemic stroke. The incidence of the carotid web is low, and it is rarely reported. Only 150 cases of FMD have been reported so far. FMD is a non-inflammatory and non-atherosclerotic arteriopathy. The most common arterial beds involved are renal and extracranial carotids. Presentation varies depending on the location of the arterial bed involved and disease severity. Clinical presentations range from minor headaches to severe headaches, resistant hypertension, acute coronary syndrome, transient ischemic attack, and in some cases, stroke. Diagnosis can be made through non-invasive methods, such as computed tomographic angiography, magnetic resonance angiography, or duplex ultrasonography or invasive imaging methods like catheter-based angiography. Treatment of FMD varies with disease presentation and its location. Asymptomatic carotid or vertebral arteries FMD should be monitored clinically and prescribed aspirin 81 mg daily for primary stroke prevention. Endovascular and surgical therapy with stents or coils is reserved for patients with aneurysms. We present a rare and interesting case of a 54-year-old female who presented with acute ischemic stroke in the setting of right carotid artery web, right internal carotid artery (ICA) thrombus with dissection, and possible pseudoaneurysm.

## Introduction

Carotid webs are a unique imaging finding that is suggestive of fibromuscular dysplasia (FMD). Arterial dissection is a common cause of stroke in the younger population; however, it can occur at any age [1]. The structural integrity of the arterial wall is compromised, resulting in dissection that leads to blood collection between the layers as an intramural hematoma. FMD is a non-specific arteriopathy that can lead to arterial stenosis, occlusion, dissection, and tortuosity. The most common arterial beds affected are renal and extracranial carotids. Presentation varies depending on the segment of arteries involved and the severity of the disease. Although it can affect individuals of all ages, it is commonly seen in middle-aged women. Patients can present in a myriad of ways, such as severe headaches, resistant hypertension, acute coronary syndrome, transient ischemic attack, and in some cases, stroke. Diagnosis can be made through invasive imaging methods, such as catheter-based angiography, or non-invasive methods, such as computed tomographic angiography (CTA), magnetic resonance angiography (MRA), or duplex ultrasonography.

## Case presentation

**Figure 1:**
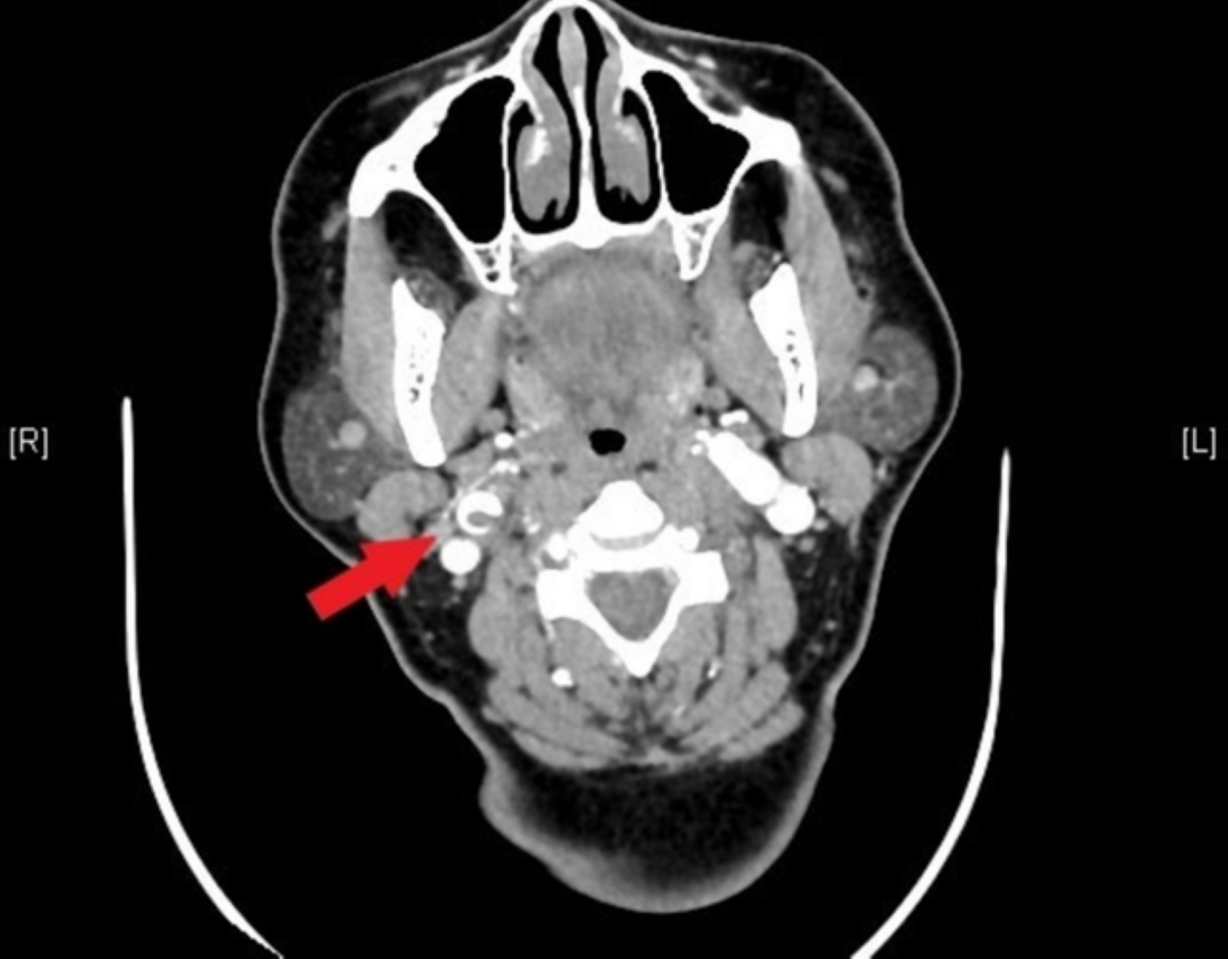
Thrombus in the right internal carotid artery

Our patient is a 54-year-old woman who came to the emergency department complaining of right-sided facial warmth and tongue paresthesias that started earlier in the day, along with an occipital headache. Her medical history was significant for hypertension (HTN), hyperlipidemia (HLD), and an ischemic stroke involving the middle and anterior cerebral arteries with residual left-sided hemiparesis. Although her symptoms resolved before arrival, emergent imaging was obtained to diagnose a possible stroke. Initial head and neck CTA showed a right internal carotid artery (ICA) thrombus with dissection and possible pseudoaneurysm (Figure 1).

**Figure 2:**
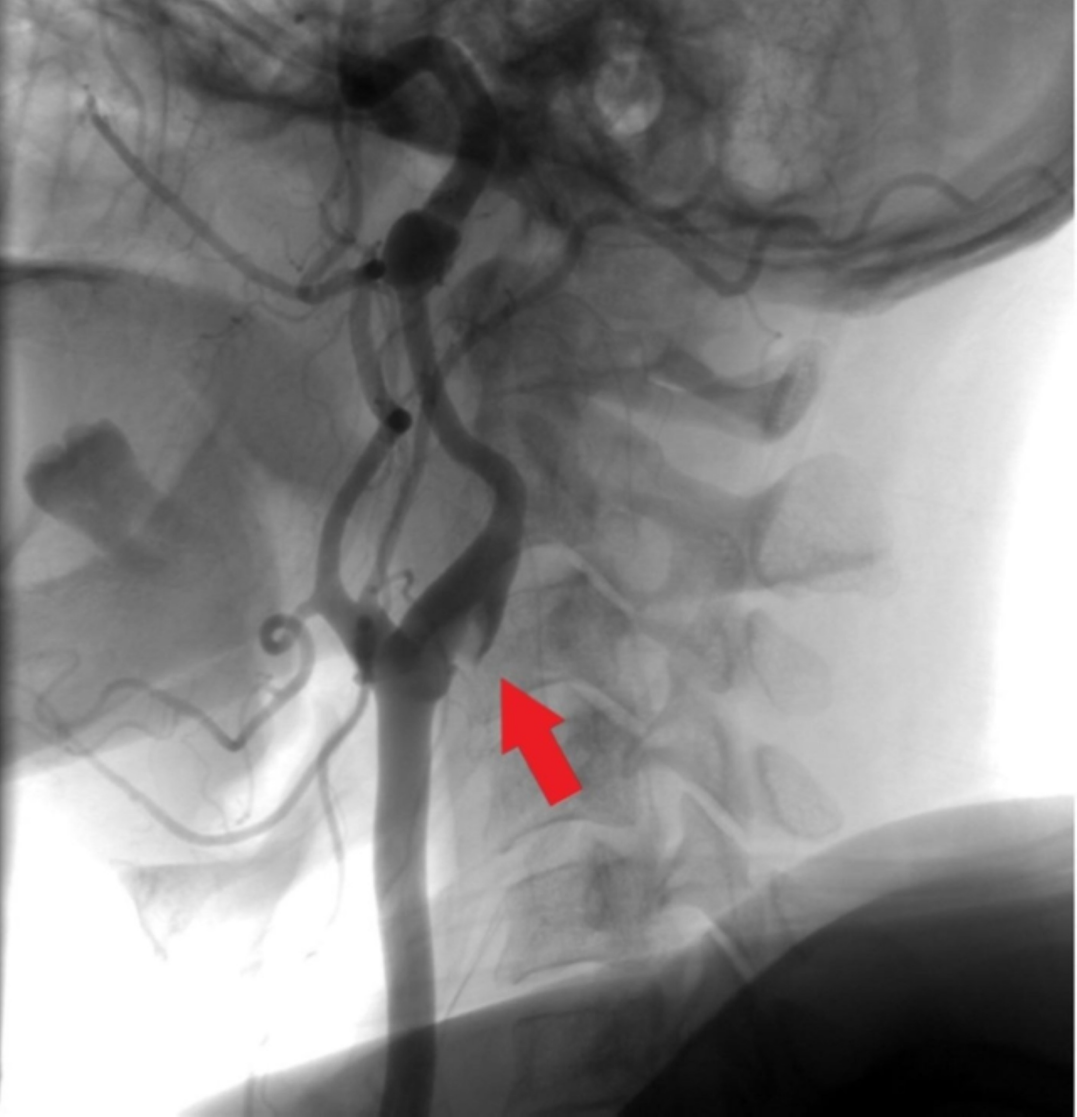
Carotid web in the right internal carotid artery

She was admitted to the ICU for further monitoring and was evaluated by neurointerventional radiology and vascular surgery. Subsequent workup included a MRI of the brain that showed a new small acute infarct in the right centrum semiovale without hemorrhagic conversion. She underwent a carotid arteriogram that showed focal irregularity and ischial-like projections from the right carotid bifurcation extending into the proximal cervical ICA, suggestive of a web reminiscent of FMD without hemodynamically significant stenosis (Figure 2).

Neurointerventional radiology recommended medical management, and the patient was treated with a heparin drip and dual antiplatelet therapy (DAPT) with aspirin and clopidogrel. Vascular surgery recommended that in the setting of recurrent episodes, a carotid endarterectomy could be entertained. She was recommended to continue DAPT for six months and to follow up with a CT arteriogram in six months. She symptomatically improved and was stable at the time of discharge.

## Discussion

Carotid webs are a unique imaging finding that is suggestive of FMD. Carotid webs are abnormal luminal projections at the carotid bulb that can be associated with blood flow stasis, artery dissection, and subsequent complications. Due to their low incidence, carotid webs are rarely reported and often underdiagnosed [1,2]. FMD is a non-atherosclerotic and non-inflammatory arteriopathy that most commonly involves the renal arteries, the extracranial carotid arteries, and the vertebral arteries. Other arteries affected by FMD include mesenteric, external iliac, and brachial arteries. Although it can affect individuals of all ages, it is commonly seen in middle-aged women. FMD was first described by Leadbetter and Burkland in 1938 and classified pathologically by Harrison and McCormick in 1971 [3,4]. Pathological specimens are rarely obtained to diagnose FMD, and pathological classification of FMD has been replaced by radiographic classification [5]. FMD is most commonly classified by angiographic appearance, and two angiographic subtypes of FMD have been reported. Multifocal FMD is the most common type and has an appearance of “string on beads” on angiography. Focal FMD is another type that appears as a "circumferential or tubular stenosis" in angiography. Carotid web can be identified in an angiogram as a linear spur-like intraluminal projection along the posterior wall of the proximal ICA on the oblique sagittal section and as a septum on axial section [6].

The etiology and pathogenesis of FMD remain unclear; however, some studies have shown an autosomal mode of inheritance with variable penetration. Most recent understanding of FMD comes from the United States Registry for FMD published in 2012 from the first 447 enrolled patients [7]. FMD involves medium-sized arteries that may lead to arterial stenosis, aneurysm, occlusion, dissection, and arterial tortuosity. Presentation varies depending on the segment of arteries involved and the severity of the disease. The most common symptoms of FMD include headache, pulsatile tinnitus, neck pain, flank pain, and abdominal pain. The most common signs of FMD include hypertension, which is mostly seen in renal artery FMD, cervical bruit, abdominal bruit, acute coronary syndromes, transient ischemic attack, and stroke. FMD should be suspected if a woman under the age of 60 years presents with hypertension, especially if severe or resistant hypertension on the presentation. The diagnosis of FMD is confirmed by diagnostic imaging. Initial imaging usually includes non-invasive tests, such as CTA, MRA, and duplex ultrasonography [8]. CTA is preferred over MRA due to higher spatial resolution, less technical dependence, and shorter scan time. Invasive imaging methods like catheter-based angiography can be used in the setting of high clinical suspicion and negative non-invasive tests. Invasive tests also serve therapeutic purposes. Treatment of FMD varies with disease presentation and its location. Asymptomatic carotid or vertebral arteries FMD should be monitored clinically and prescribed aspirin 81 mg daily for primary stroke prevention. In the setting of arterial thrombus occlusion, medical treatment includes DAPT and anticoagulation therapy. Percutaneous transluminal balloon angioplasty (PTA) is the first line of treatment for those with symptomatic extracranial cerebral vascular FMD. Endovascular and surgical therapy with stents or coils is reserved for patients with aneurysms [9]. In renal artery FMD, the main goal is strict blood pressure control to prevent sequelae of long-standing poorly controlled HTN. In patients with newly diagnosed HTN secondary to FMD, initial treatment includes PTA [10]. For patients with long-standing HTN where renal FMD was not diagnosed at the onset of HTN, antihypertensive medications should be continued. Patients should be monitored with close clinical follow-up with blood pressure measurement and a renal function test every six months. Renal artery ultrasound should be repeated every 6-12 months.

## Conclusions

Carotid webs are a unique imaging finding that is suggestive of FMD. We present a rare and interesting case of a 54-year-old female with the right carotid web who presented with acute ischemic stroke with the right ICA thrombus with dissection and possible pseudoaneurysm. FMD is a non-specific arteriopathy that can lead to arterial stenosis, occlusion, dissection, and tortuosity. The most common arterial beds affected are renal and extracranial carotids. Presentation varies depending on the segment of arteries involved and the severity of the disease. The history and the clinical presentation are important as FMD presents in a wide range of symptoms. Undiagnosed cases have more morbidity and mortality. Although diagnostic criteria are outlined well, a rare presentation of these conditions poses a significant challenge for a clinician.

## References

[1] Kawahara I, Hiu T, Ono T, Haraguchi W, Ushijima R, Tsutsumi K (2019). Reconsideration of carotid web and the therapeutic strategy (Article in Japanese). No Shinkei Geka.

[2] Zhang AJ, Dhruv P, Choi P (2018). A systematic literature review of patients with carotid web and acute ischemic. Stroke.

[3] Leadbetter WF, Burkland CE (1938). Hypertension in unilateral renal disease. J Urol.

[4] Harrison EG Jr, McCormack LJ (1971). Pathologic classification of renal arterial disease in renovascular hypertension. Mayo Clin Proc.

[5] Stanley JC, Gewertz BL, Bove EL, Sottiurai V, Fry WJ (1975). Arterial fibrodysplasia: histopathologic character and current etiologic concepts. Arch Surg.

[6] Choi PM, Singh D, Trivedi A (2015). Carotid webs and recurrent ischemic strokes in the era of CT angiography. AJNR Am J Neuroradiol.

[7] Olin JW, Froehlich J, Gu X (2012). The United States Registry for fibromuscular dysplasia: results in the first 447 patients. Circulation.

[8] Lewis S, Kadian-Dodov D, Bansal A, Lookstein RA (2016). Multimodality imaging of fibromuscular dysplasia. Abdom Radiol.

[9] Tekieli ŁM, Maciejewski DR, Dzierwa K (2015). Invasive treatment for carotid fibromuscular dysplasia. Postepy Kardiol Interwencyjnej.

[10] Meuse MA, Turba UC, Sabri SS, Park AW, Saad WEA, Angle JF, Matsumoto AH (2010). Treatment of renal artery fibromuscular dysplasia. Tech Vasc Interv Radiol.

